# Successful Cancer Surgery Without Transfusion Following Early Discontinuation of Dual Antiplatelet Therapy After Percutaneous Coronary Intervention for Acute Myocardial Infarction

**DOI:** 10.3390/jcm14186456

**Published:** 2025-09-13

**Authors:** Sungmin Suh, Nayoung Kim, Sangho Kim

**Affiliations:** Department of Anesthesia and Pain Medicine, Soonchunhyang University Seoul Hospital, Seoul 04401, Republic of Korea; suh5701@schmc.ac.kr (S.S.); nagau1995@gmail.com (N.K.)

**Keywords:** acute myocardial infarction, Jehovah’s witness, acute normovolemic hemodilution, antiplatelet therapy, neoplasms/surgery

## Abstract

A 75-year-old Jehovah’s Witness with recent ST-elevation myocardial infarction (STEMI) underwent percutaneous coronary intervention (PCI) with stenting of the proximal LAD. She was later diagnosed with gallbladder cancer and required urgent surgery but firmly refused allogeneic blood transfusion. This posed a major challenge, as the surgery was expected to cause significant bleeding, and the patient had undergone coronary stenting within the previous three months, which is when the risk of stent thrombosis is highest if dual antiplatelet therapy (DAPT) is interrupted. After conducting a careful multidisciplinary discussion and obtaining informed consent, both aspirin and clopidogrel were discontinued five days preoperatively. Through comprehensive blood conservation strategies—including acute normovolemic hemodilution (ANH), intraoperative cell salvage, and robotic-assisted minimally invasive surgery—the patient successfully underwent extended cholecystectomy without transfusion. This case highlights the possibility of safe, completely transfusion-free major surgery in patients with recent PCI and high thrombotic risk when individualized perioperative planning is applied.

## 1. Introduction

Blood transfusion plays a crucial role in maintaining hemodynamic stability and ensuring adequate oxygen delivery to tissues during the intraoperative management of surgeries associated with a high risk of bleeding. However, in patients who refuse transfusion for religious or personal reasons—such as Jehovah’s Witnesses—managing intraoperative bleeding is highly challenging. The continuous administration of crystalloids or colloids alone can exacerbate anemia, compromising oxygen delivery at the tissue level. To address this, various blood conservation strategies are employed in transfusion-free surgeries, especially when bleeding is anticipated. These include the use of antifibrinolytics, acute normovolemic hemodilution (ANH), intraoperative cell salvage, and minimally invasive surgical techniques, which all aim to minimize blood loss and maintain hemoglobin levels without transfusion [1,2,3,4,5,6].

ST-segment elevation myocardial infarction (STEMI), a severe form of acute coronary syndrome (ACS), demands prompt diagnosis and intervention due to its high morbidity and mortality. Primary percutaneous coronary intervention (PCI), followed by dual antiplatelet therapy (DAPT), significantly improves survival outcomes and is the cornerstone of STEMI management. The 2025 ACC/AHA and 2023 ESC guidelines recommend continuing DAPT for at least 12 months after PCI (Class I), with a possible reduction to 6 months in patients at high risk of bleeding [7,8]. Despite advances in drug-eluting stent (DES) technology, stent thrombosis remains a catastrophic complication, particularly when DAPT is discontinued prematurely—most critically within the first month of stent implantation [9,10,11]. Certain clinical circumstances, such as the need for urgent non-cardiac surgery, may necessitate the early interruption of DAPT, placing clinicians in a difficult position where they have to balance the risks of thrombosis and bleeding. Although some studies have addressed early DAPT cessation in surgical settings, very few cases involve patients who simultaneously face a high thrombotic risk and strictly refuse transfusion [9,10,11,12].

We present a rare case of urgent oncologic surgery performed within 3 months of PCI, in which both aspirin and clopidogrel were discontinued, and the surgery was successfully performed without complications or allogeneic transfusion by employing multimodal blood conservation strategies. Through a combination of ANH, intraoperative cell salvage, and robot-assisted surgery, successful outcomes were achieved. This case highlights the feasibility and importance of individualized perioperative strategies for complex patients requiring both ischemic risk mitigation and blood conservation.

## 2. Case Report

A 75-year-old female patient (68.5 kg, 155.5 cm) presented to an outside hospital on 22 June 2025, with the onset of abdominal pain. A CT scan was performed, which revealed gallbladder (GB) cancer with hemobilia and GB distension without evidence of active bleeding. Surgical intervention was deemed necessary. However, the patient was a Jehovah’s Witness and refused allogeneic blood transfusions. She had a medical history of hypothyroidism and a recent ST-elevation myocardial infarction (STEMI) due to a 90% stenosis of the proximal left anterior descending artery (pLAD), for which she underwent percutaneous coronary intervention (PCI) with stent placement on 24 May 2025. As such, she required continued dual antiplatelet therapy (DAPT).

At the referring hospital, the cardiology team advised that elective surgery would be safest after 12 months of uninterrupted DAPT. If earlier surgery was essential, then they recommended continuing aspirin while discontinuing clopidogrel for 5 days, provided that at least 3 months had passed since the PCI. Given that the PCI had been performed within the previous 3 months, the complete perioperative interruption of antiplatelet therapy posed a significant risk of thrombosis. Furthermore, due to the patient’s refusal of transfusion, the risk of bleeding was a critical consideration. The initial hospital concluded that surgery could not be safely performed without transfusion support and referred the patient to our center, which has experience in bloodless surgery.

Upon transfer, percutaneous transhepatic gallbladder drainage (PTGBD) was first performed on 2 July 2025. A subsequent preoperative evaluation revealed the following: hemoglobin (Hb) at 9.5 g/dL, hematocrit (Hct) at 29.8%, a platelet count of 338,000/μL, a normal coagulation profile, normal thyroid function (free T4 and TSH), and normal cardiac biomarkers (troponin T < 0.003 ng/mL). Liver function tests showed the following: AST/ALT 105/67 IU/L, ALP 257 IU/L, GGT 228 IU/L, total bilirubin 0.7 mg/dL, and albumin 3.7 g/dL. Transthoracic echocardiography (TTE) conducted at the initial facility on 26 May 2025, revealed a normal left ventricular ejection fraction. The baseline electrocardiogram (ECG) showed sinus rhythm with a heart rate of 55 bpm, an incomplete right bundle branch block, QT prolongation (QT/QTc 493/474 ms), negative T waves, and slight left axis deviation. A multidisciplinary consultation was held, which included the cardiology team, who reiterated the general recommendation for 12 months of DAPT post-PCI. Nonetheless, given the urgent need to prevent cancer progression and the high risk of bleeding associated with the procedure, our cardiology team agreed that surgery could proceed with the discontinuation of all antiplatelet agents if the patient and family consented after being fully informed. Informed consent was obtained, and surgery was scheduled. The patient had been taking dual antiplatelet therapy consisting of aspirin and clopidogrel, both of which were discontinued five days prior to surgery. Thus, surgery was ultimately performed on 23 July 2025, exactly 60 days after the PCI, following a course of DAPT.

On 23 July 2025, the patient entered the operating room with an 18G intravenous (IV) line in the left leg. Standard monitoring was applied, including a 5-lead ECG with ST-segment analysis, pulse oximetry, non-invasive blood pressure, bispectral index (BIS), and neuromuscular monitoring. After local anesthesia, a left radial arterial line was cannulated and connected to FloTrac for continuous hemodynamic monitoring. General anesthesia was induced using 40 mg of lidocaine, 100 mg of propofol, 50 mg IV bolus of rocuronium, and continuous remifentanil infusion at 0.04 mcg/kg/min. Maintenance was achieved with remifentanil and sevoflurane. Following intubation, the IV line in the left leg was removed, and a new 18G IV line was placed in the right arm. To optimize surgical field visualization, a Levin tube (L-tube) was inserted after endotracheal intubation. A right internal jugular central venous catheter was placed without complications, and central venous pressure (CVP) monitoring commenced.

Before surgery, 200 mL of Volulyte was infused, and ANH was performed via the arterial line, collecting 400 mL of blood over 30 min. The initial arterial blood gas analysis revealed 10.6 g/dL of Hb and normal electrolytes. Intraoperatively, a continuous infusion of rocuronium at 4.8 mcg/kg/min maintained deep neuromuscular blockade. Hemodynamic stability was supported with norepinephrine infusion via the central line to maintain a target MAP > 65 mmHg.

The patient underwent a robot-assisted single-port extended cholecystectomy with lymph node dissection and wedge resection of the liver around the GB. The total anesthesia time was 7 h, and the surgical duration was 5 h and 25 min. The estimated blood loss was minimal at 50 mL. As intraoperative bleeding was limited, only a small amount of blood was collected through the cell saver, and reinfusion was not necessary. The autologous blood obtained from ANH was reinfused before the end of surgery.

**Figure 1:**
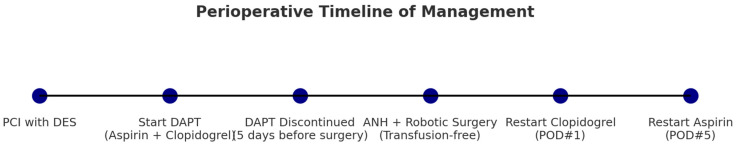
Perioperative timeline of patient management.

At the end of the procedure, the patient was reversed with sugammadex and extubated after the administration of 100 mcg of fentanyl. She was then transferred to the surgical ICU in stable condition. Immediate postoperative Hb was 10.2 g/dL, and Hct was 31.4%. On postoperative day 1 (POD#1), Hb was 9.5 g/dL, and Hct was 28.9%, with minimal drain output (10 mL at POD#1) and a good general condition. Clopidogrel was resumed on POD#1, and the patient was transferred to the general ward. Aspirin was re-initiated on POD#5 without complications. The overall clinical timeline from PCI to the postoperative resumption of aspirin is illustrated in Figure 1.

Histopathological examination confirmed gallbladder adenocarcinoma, moderately differentiated. At the outpatient follow-up with general surgery on 26 August 2025, the patient was stable. On 27 August 2025, she visited the oncology outpatient clinic, where adjuvant capecitabine chemotherapy was recommended; however, the patient declined. A plan was made for further follow-up in 3 months.

## 3. Discussion

Managing patients who require urgent non-cardiac surgery shortly after percutaneous coronary intervention (PCI) with drug-eluting stent (DES) placement presents a significant clinical dilemma, particularly when compounded by patient-specific factors such as refusal of transfusion. In this case, a 75-year-old Jehovah’s Witness patient underwent robot-assisted cancer surgery without transfusion following the complete discontinuation of dual antiplatelet therapy (DAPT) within 3 months of PCI—a scenario rarely documented in the literature.

Current guidelines from the 2025 American Heart Association/American College of Cardiology (AHA/ACC) and the 2023 European Society of Cardiology (ESC) recommend maintaining DAPT for at least 12 months following PCI in patients with ST-elevation myocardial infarction (STEMI), citing Class I evidence. In patients at high risk of bleeding, a reduced duration of 6 months may be considered. However, premature cessation of DAPT—especially within the first month—is strongly associated with stent thrombosis and adverse cardiac events, making such decisions highly consequential [7,8,9,10,11,12].

In our case, both aspirin and clopidogrel were stopped five days preoperatively, despite the PCI being performed less than 3 months prior. This decision deviated from standard guidelines and was not taken lightly. The patient’s refusal of transfusion meant that even moderate bleeding could have catastrophic consequences. For this reason, absolute hemostatic control was prioritized, and the complete discontinuation of antiplatelet therapy was deemed the safest strategy to minimize the risk of intraoperative bleeding. This decision was reached after conducting a thorough multidisciplinary discussion and obtaining full informed consent from the patient and her family.

Another important consideration is the hypercoagulable state associated with malignancy. Cancer induces systemic prothrombotic changes through platelet activation, endothelial dysfunction, and inflammatory mediators, which collectively increase the risk of both arterial and venous thromboembolism [13]. Beyond venous events, cancer patients also exhibit a significantly higher risk of arterial thrombosis than the general population [14]. Mechanistically, tumor cells secrete prothrombotic mediators such as thrombin and vascular endothelial growth factor (VEGF), which enhance platelet activation and coagulation and may promote arterial thrombosis even in the absence of atherosclerotic plaque [15]. These cancer-specific mechanisms contribute to an intrinsically hypercoagulable state that amplifies ischemic risk in patients with coronary stents [13,15]. Against this background, stopping DAPT only two months after PCI placed the patient at a substantially elevated risk of stent thrombosis, further magnifying the complexity of perioperative decision-making in this case.

To mitigate hemorrhagic risk, the anesthesia and surgical teams employed multimodal blood conservation strategies, including acute normovolemic hemodilution (ANH), careful hemodynamic monitoring using FloTrac, and vasopressor support to maintain perfusion while minimizing bleeding. The use of minimally invasive robotic surgery further contributed to the low estimated blood loss of 50 mL. These interventions collectively enabled the patient to undergo high-risk cancer surgery without transfusion.

In addition to minimizing perioperative transfusion requirements, ANH has been associated with broader clinical and physiological benefits. A retrospective study of major oncologic surgery patients demonstrated that ANH was independently associated with a reduced length of hospital stay [16]. Experimental studies also suggest that ANH improves microcirculation and tissue oxygenation in ischemic and hypoxic models [17]. Furthermore, in anesthetized patients with coronary artery disease, ANH was shown to preserve left ventricular systolic and diastolic function while decreasing blood viscosity, thereby augmenting venous return, cardiac preload, and stroke volume [18]. These findings reinforce the rationale for applying ANH in oncologic patients with adequate baseline hematocrit, highlighting its potential role in enhancing tissue oxygenation, maintaining cardiac function, and supporting faster recovery.

The timing of antiplatelet resumption was another critical component of perioperative management. Clopidogrel was restarted on postoperative day 1, while aspirin was re-initiated on day 5. This approach was based on an individualized multidisciplinary assessment rather than strict institutional protocols. The cardiology team emphasized minimizing the duration of complete DAPT interruption, whereas the surgical team prioritized confirmation of hemostasis and stable drain output before reintroducing aspirin. Recent evidence has shown that in-hospital bleeding among acute coronary syndrome (ACS) patients significantly affects the continuation of post-discharge therapies and long-term prognosis [19]. In particular, a recent study reported that patients with STEMI, female sex, and advanced age—similar to the profile of our patient—were more prone to in-hospital bleeding events. Moreover, the occurrence of in-hospital bleeding was strongly associated with adverse outcomes, including increased all-cause mortality [19]. These findings underscore the importance of carefully balancing thrombotic protection with bleeding risk and tailoring antiplatelet resumption strategies in vulnerable subgroups, such as that of our patient.

In the context of bloodless surgery for Jehovah’s Witness patients, several strategies have been widely studied, including preoperative blood augmentation and anemia management, intraoperative ANH and cell salvage, and efforts to minimize intraoperative bleeding such as minimally invasive surgery [4,5,6]. Our case shared common elements with this paradigm, as intraoperative ANH and a cell saver system were employed, while the robotic surgical approach further contributed to reducing the risk of bleeding.

However, unlike some reports that used preoperative erythropoiesis-stimulating strategies with intravenous iron and recombinant erythropoietin (EPO) to increase hemoglobin levels, we deliberately avoided EPO in this patient. Previous evidence has shown that EPO use in cancer-associated anemia can increase the risk of venous thromboembolism and mortality [20]. Given that cancer itself confers a hypercoagulable state and that our patient was already at heightened risk of stent thrombosis due to a recent PCI and DAPT interruption, the risks outweighed the potential benefits.

Similarly, antifibrinolytic therapy with tranexamic acid, which has been applied in both cardiac [6] and non-cardiac [21] surgical settings to reduce perioperative bleeding, was not utilized in our case. The POISE-3 trial demonstrated reduced bleeding but also raised concerns about thrombotic complications, and, in the setting of malignancy and recent stenting, we considered this an unacceptable additional risk.

Overall, our strategy aligns with the broader literature on bloodless oncologic and cardiovascular surgery [4,5,6,20,21] but also illustrates the necessity of tailoring blood conservation approaches to the individual thrombotic risk profile. By integrating ANH, cell salvage, and robotic minimally invasive surgery—while avoiding agents that could exacerbate thrombosis—we successfully navigated the competing risks of bleeding and stent thrombosis in this complex patient.

This case exemplifies the importance of individualized perioperative care in complex and ethically challenging situations. While guidelines provide a framework for standard practice, clinical judgment, patient autonomy, and multidisciplinary collaboration are paramount when deviations from recommended protocols are necessary. Our experience highlights how rigorous perioperative planning and respect for patient values can be harmonized to achieve favorable outcomes, even in high-risk transfusion-free surgery. At the same time, further studies and multicenter registries are warranted to establish evidence-based protocols, particularly regarding optimal perioperative antiplatelet management, the timing of surgery, and the role of patient blood management programs in such high-risk patients.

## Data Availability

The datasets generated or analyzed during this study are available from the corresponding author on reasonable request.

## Abbreviations

The following abbreviations are used in this manuscript:

STEMI	ST-elevation myocardial infarction
PCI	Percutaneous coronary intervention
pLAD	Proximal left anterior descending artery
DAPT	Dual antiplatelet therapy
ANH	Acute normovolemic hemodilution
DES	Drug-eluting stent
